# Insulin Promotes the Proliferation of Human Umbilical Cord Matrix-Derived Mesenchymal Stem Cells by Activating the Akt-Cyclin D1 Axis

**DOI:** 10.1155/2017/7371615

**Published:** 2017-04-18

**Authors:** Peng Li, Jinsong Wei, Xiang Gao, Bo Wei, Hao Lin, Rui Huang, Yanru Niu, Kyu Lim, Kaipeng Jing, Jiaqi Chu

**Affiliations:** ^1^Stem Cell Research and Cellular Therapy Center, Affiliated Hospital of Guangdong Medical University, Zhanjiang, China; ^2^Department of Spinal Surgery, Affiliated Hospital of Guangdong Medical University, Zhanjiang, China; ^3^Laboratory Institute of Minimally Invasive Orthopedic Surgery, Affiliated Hospital of Guangdong Medical University, Zhanjiang, China; ^4^Department of Biochemistry, School of Medicine, Chungnam National University, Daejeon, Republic of Korea

## Abstract

*Background*. The functions of insulin in mesenchymal stem cells (MSC) remain poorly understood. *Methods.* MSC from human umbilical cord matrix (UCM) cultured in serum-free media (SFM) with or without insulin were subjected to various molecular biological analyses to determine their proliferation and growth states, expression levels of Akt-cyclin D1 signaling molecules, and in vitro differentiation capacities. *Results.* Insulin accelerated the G1-S cell cycle progression of UCM-MSC and significantly stimulated their proliferation and growth in SFM. The pro-proliferative action of insulin was associated with augmented cyclin D1 and phosphorylated Akt expression levels. Akt inactivation remarkably abrogated insulin-induced increases in cyclin D1 expression and cell proliferation, indicating that insulin enhances the proliferation of UCM-MSC via acceleration of the G1-S transition mediated by the Akt-cyclin D1 pathway. Additionally, the UCM-MSC propagated in SFM supplemented with insulin exhibited similar specific surface antigen profiles and differentiation capacities as those generated in conventional media containing fetal bovine serum. *Conclusions.* These findings suggest that insulin acts solely to promote UCM-MSC proliferation without affecting their immunophenotype and differentiation potentials and thus have important implications for utilizing insulin to expand clinical-grade MSC in vitro.

## 1. Introduction

Mesenchymal stem cells (MSC) were originally obtained as a fibroblast-like subset of stromal cells in the bone marrow (BM) and have since been isolated from virtually all postnatal tissues [1]. Although a few differences such as doubling times exist among MSC isolated from various adult tissues, they are widely defined using minimal criteria based on their propensity to adhere to plastic, absence of CD34, CD45, and CD14, and expression of CD73, CD90, and CD105 and ability to differentiate into adipocytes, osteoblasts, and chondrocytes in vitro [2]. As MSC possess self-renewal and multipotent differentiation potentials, these cells have been proposed as a promising candidate for tissue engineering and cell therapy [3]. BM-MSC are the most extensively studied population of MSC; however, it is becoming increasingly clear that MSC of neonatal origins, in particular those derived from umbilical cord matrix (UCM), may represent a more suitable population than BM-MSC for clinical use due to their noninvasive harvest procedure, shorter doubling time, and greater long-term growth ability [4].

The frequency of MSC residing within human tissues is rather low, and thus, primary MSC necessitate in vitro expansion to yield sufficient numbers (approximately 1–4 × 10^6^ MSC/kg per infusion) prior to clinical applications [5]. This is conventionally achieved in medium containing fetal bovine serum (FBS), which may carry infectious agents from animals and initiate xenogeneic immune responses following MSC transplantation [6]. Likewise, FBS has a considerable degree of interbatch variation, leading to wide variation in its capacity to support MSC expansion even under the same culture conditions [7]. Therefore, serum-free strategies using exogenous growth factors have been proposed to implement the clinical-scale production of MSC [8].

Insulin is a secreted peptide hormone whose primary role is to regulate the blood glucose level at the whole organism level. Meanwhile, although inconsistent results exist in the literature [9, 10], several studies revealed that insulin can promote the proliferation of different cell types [11, 12], indicating that it also possesses properties of tissue growth factors. Via binding to its membrane receptor, insulin can enhance cell division by modulating various cellular signaling components [13-15]. For instance, the positive effect of insulin on the proliferation of human epithelial cells and hamster ovary cells is directly associated with the protein kinase Akt and extracellular signal-regulated kinase (ERK), two key regulators of cell cycle progression [16, 17].

In the present study, the proliferative capacity, specific surface antigens, and differentiation potential of human UCM-MSC cultured in insulin-supplemented serum-free media were determined. We found that insulin promoted UCM-MSC proliferation under this condition, without influencing their multilineage potentiality and immunophenotype. Further experiments revealed that activation of the Akt-cyclin D1 axis was responsible for the pro-proliferative effect of insulin. These results lead to a better understanding of how insulin affects MSC and provide evidence for the use of insulin in serum-deficient media for propagation of clinical-grade MSC.

## 2. Methods

### 2.1. Antibodies and Reagents

The phosphorylated Akt (Ser 473) and ERK (Thr202/Tyr204) antibodies were obtained from Cell Signaling Technology (Beverly, MA, USA, #9271 and #4370), and the antibodies against cyclin D1 (#SC-718), glyceraldehyde-3-phosphate dehydrogenase (GAPDH, #SC-365062), and horseradish peroxidase-conjugated goat anti-rabbit/mouse secondary antibodies (#SC-2004 and #SC-2005) were purchased from Santa Cruz Biotechnology (Santa Cruz, CA, USA). Insulin solution and an Akt inhibitor, LY294002, dissolved in dimethyl sulfoxide were from Sigma-Aldrich (St. Louis, MO, USA, #I0516) and Merck Millipore (San Diego, CA, USA, #440202), respectively.

### 2.2. Cell Culture and Treatment

Primary UCM-MSC at passage 2 from healthy full-term and naturally delivered newborns were purchased from Cyagen (Guangzhou, China, #HUXUC-01001). These cells were expanded in normal serum-containing media (SCM) consisting of Dulbecco's modified Eagle's medium (DMEM, #10569), 10% FBS (#10082), and 1% penicillin-streptomycin (#15140) (all from Thermo Fisher Scientific, Grand Island, NY, USA) and incubated in a humidified 5% CO_2_ atmosphere at 37°C. The cells were passaged twice weekly, and experiments were performed with the subcultured cells between passages 3 and 6. Unless otherwise stated, UCM-MSC were plated onto either 6 or 10 cm dishes at 70% confluence and allowed to attach overnight in the SCM. The cells were washed once with phosphate-buffered saline (PBS) and then exposed to various compounds in serum-free medium (SFM, DMEM with 1% penicillin-streptomycin) for 72 h.

### 2.3. Cell Proliferation Assay

To assess the effect of insulin and/or Akt inactivation on cell proliferation, UCM-MSC were seeded onto 96-well plates (2000 cells/well). The cells, after overnight attachment in SCM, were washed with PBS once and then switched into 200 *μ*L of SFM supplemented with different doses of insulin in the absence or presence of 5 *μ*M LY294002 for 72 h. The proliferation of UCM-MSC was determined using cell-counting kit-8 (CCK-8, Beyotime Biotechnology, Shanghai, China, #C0038) according to the manufacturer's instructions. Briefly, at the end of treatment, CCK-8 solution (20 *μ*L) was added to each well, followed by incubation for 1 h at 37°C. The absorbance at 450 nm was determined by a SpectraMax M5 microplate reader (Molecular Devices, Sunnyvale, CA, USA). Cell proliferation was expressed as a percentage relative to the untreated cells. For each group, mean values of the absorbance from six wells were calculated.

### 2.4. Phenotypic Characterization of UCM-MSC

For comparison of surface marker expression between UCM-MSC expanded in SCM and those expanded in SFM supplemented with insulin, cells grown under abovementioned conditions for one passage were harvested; stained using antibodies for human CD34-phycoerythrin (PE), CD105-PE, CD31-fluorescein isothiocyanate (FITC), CD90-FITC, and CD45-allophycocyanin (APC); and analyzed by a FACSCanto II flow cytometer (all from BD Biosciences, San Jose, CA, USA) following the manufacturer's instructions. The background fluorescence levels were set using corresponding isotype controls, and the FlowJo software (Treestar, Ashland, OR, USA) was employed to analyze the collected data.

### 2.5. Morphometry

Cell morphology was inspected and photographed using an EVOS™ XL Core Cell Imaging System (Thermo Fisher Scientific). Biological variables of the cells, including size and complexity, were obtained by measuring the forward scatter (FSC) and side scatter (SSC) parameters, respectively. In this case, UCM-MSC with or without insulin stimulation were washed twice with PBS at the end of experiments, harvested with trypsin, and kept on ice until FSC and SSC were measured by flow cytometry in the FACSCanto II device. For measurement, a region based on FSC (FSC-R1) or SSC (SSC-R2) properties, which included approximately 75% of events, was first set separately in the FSC versus SSC dot plot of control cells and then applied to the dot plot of cells treated with insulin, followed by comparison of the percentage of cells gated in each region. A reduction in the percentage of cells gated in FSC-R1 or SSC-R2 reflects an increased cell population with larger cell size and more internal cellular complexity, respectively, and vice versa.

### 2.6. Differentiation Analysis

After culturing in SCM or SFM supplemented with 10 *μ*M insulin for one passage, UCM-MSC were reseeded onto 12-well plates (2 × 10^5^ cells/well) and allowed to reach confluency in SCM. The cells were then induced to differentiate into adipocytes and osteoblasts, and the differentiation capacity was measured by staining with oil red O or alizarin red S using Oricell™ differentiation kits (Cyagen, #HUXUC-90031 and #HUXUB-90021) following the manufacturer's instructions. For quantification of the deposited oil red O or alizarin red S, the dye was extracted from cells using 600 *μ*L of absolute ethanol or 10% cetylpyridinium chloride solution for 1 h at room temperature with gentle shaking, aliquots of 150 *μ*L were transferred to a 96-well plate in triplicate, and absorbance was then measured at 450 and 560 nm, respectively, using the SpectraMax M5 microplate reader.

### 2.7. Western Blotting

UCM-MSC were lysed using RIPA buffer (Thermo Fisher Scientific, #89900) containing protease inhibitor cocktail (Solarbio, Beijing, China, #P1260). Protein concentrations were determined using BCA protein assay kit (Beyotime Biotechnology, #P0012). Total protein (30 *μ*g) was resolved by 10–12% SDS-PAGE gels and then transferred to polyvinylidene difluoride membranes (Merck Millipore, #ISEQ00010). The membranes were then blocked in 5% (*w*/*v*) skimmed milk for 1 h and incubated with primary antibodies against phosphorylated Akt, phosphorylated ERK, cyclin D1, and GAPDH overnight at 4°C, followed by application of secondary antibodies. The blots were developed using a commercially available enhanced chemiluminescence (Merck Millipore, #WBKLS0100). Quantification of band intensity was performed using the GS 800 densitometer and Quantity One software (both from Bio-Rad Laboratories, Hemel Hempstead, UK). The results were normalized to GAPDH protein level and expressed as a fold change over the untreated control group.

### 2.8. Cell Cycle Analysis

UCM-MSC cultured in SFM supplemented with or without insulin were harvested and fixed in 70% ethanol at −20°C overnight. After fixation, the cells were washed three times with PBS, resuspended in PBS containing 100 *μ*g/mL of RNase A (Solarbio, #R1030) for 30 min, and then treated with 50 *μ*g/mL propidium iodide (PI, Sigma-Aldrich, #P4170) for 10 min in the dark. Subsequently, the samples were acquired in the FACSCanto II system and the percentage of cells in each phase of the cell cycle was determined using the Watson modeling algorithm from the FlowJo software. Three independent biological replicates were prepared for each condition, and at least 10000 events per sample were acquired.

### 2.9. Statistical Analysis

Comparisons were calculated using one-way analysis of variance (ANOVA) or Student's *t*-test wherever applicable. Results are shown as mean ± SD values, with a minimum of three separate experiments for each issue addressed. *p* values of less than 0.05 were considered significant.

## 3. Results

### 3.1. Insulin Promotes UCM-MSC Proliferation and Growth in SFM

**Figure 1:**
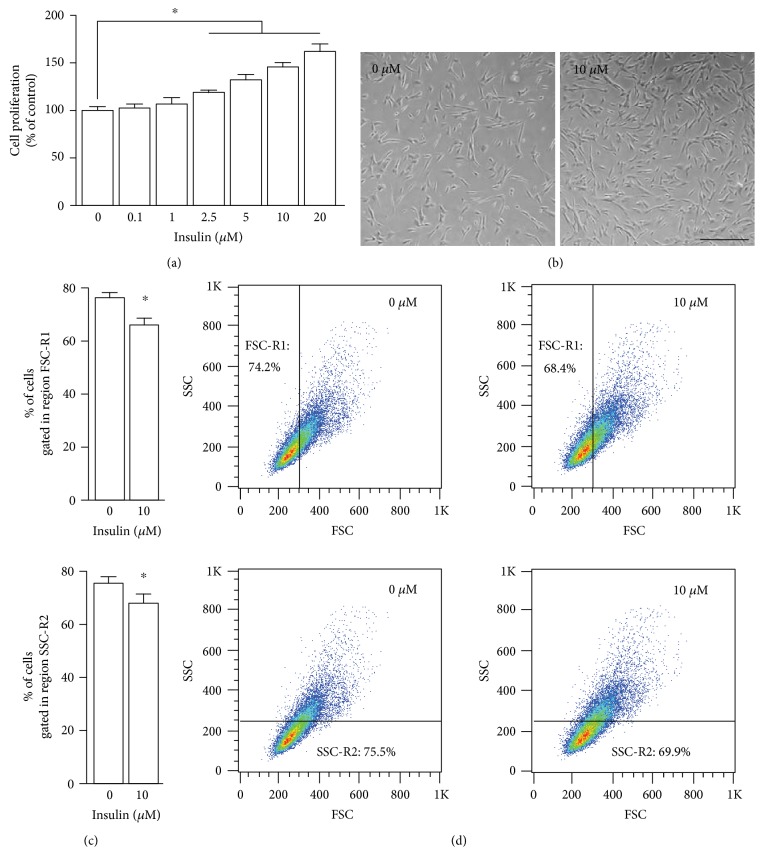
Effects of insulin on the proliferation, morphology, and growth of UCM-MSC in SFM. (a) Proliferation of UCM-MSC cultured in SFM without or with insulin. UCM-MSC were incubated in SFM supplemented with increasing dosages of insulin (0–20 *μ*M) for 72 h, and proliferation of UCM-MSC was measured by CCK-8 assays. Values are adjusted relative to the proliferation of the nontreatment control, which was set to 100%. (b) Photomicrograph of UCM-MSC cultured in SFM for 72 h with or without 10 *μ*M insulin (scale bar, 100 *μ*m). (c, d) UCM-MSC incubated in SFM containing 0 and 10 *μ*M insulin for 72 h were subjected to flow cytometry, and the cell size and internal complexity were analyzed as described in Section 2.5. Bar graphs (c) show the results of three separate experiments and represent the difference in size (top, FSC-R1) and internal complexity (bottom, SSC-R2). Two-dimensional plots (d) show a representative experiment. All data are represented as mean ± SD, and error bars indicate SD (*n* = 3). ^∗^*p* < 0.05 versus nontreated control cells by one-way ANOVA with Fisher's test for (a) and Student's *t*-test for (c).

Since insulin enhances the proliferation of several cell types [11, 12, 14], we asked whether insulin can act as a potential mediator to sustain UCM-MSC proliferation. To investigate this, UCM-MSC were exposed to various concentrations of insulin and their proliferation activity was measured. This assay was conducted in SFM to avoid the interference of unknown amounts of insulin and growth factors present in the serum. Insulin had no or slight effects on UCM-MSC at concentrations up to 1 *μ*M, but, at concentrations of 2.5 *μ*M or higher, it significantly promoted cell proliferation in a dose-dependent manner (Figure 1(a)). Further morphological analysis showed that, consistent with the results obtained from the proliferation assay, the number of cells grown in SFM supplemented with insulin was dramatically higher than the number of cells cultured in SFM alone, and the typical fibroblast-like appearance of UCM-MSC was not altered in response to insulin treatment (Figure 1(b)). Likewise, compared with control cells, the size and internal complexity of insulin-treated UCM-MSC increased slightly, but significantly, as revealed by the reduction in the cell populations with lower FSC and SSC (Figures 1(c) and 1(d)). Meanwhile, to further evaluate the effect of insulin on MSC proliferation and to investigate whether MSC would survive in long term in SFM supplemented with insulin, MSC cultured in SFM, SCM, and SFM with increasing dosages of insulin were allowed to grow for up to 12 days and the cell proliferation was determined every 3 days (Supplementary Figure 1 available online at https://doi.org/10.1155/2017/7371615). We found that whereas the number of cells grown in SFM gradually declined within the first 6 days of culture and then kept unchanged, addition of insulin into SFM remarkably increased the MSC number over the whole testing period. The SCM also resulted in a marked increase in MSC proliferation, and this pro-proliferative effect of SCM was approximately three times as strong as that of SFM supplemented with 10 *μ*M insulin. These findings together suggest that insulin at relatively high concentrations (higher than 2.5 *μ*M), although not as effective as FBS, enhances the proliferation and growth of UCM-MSC under serum-deficient conditions.

### 3.2. The Pro-Proliferative Effect of Insulin on UCM-MSC Involves Cyclin D1-Mediated G1-S Phase Progression

**Figure 2:**
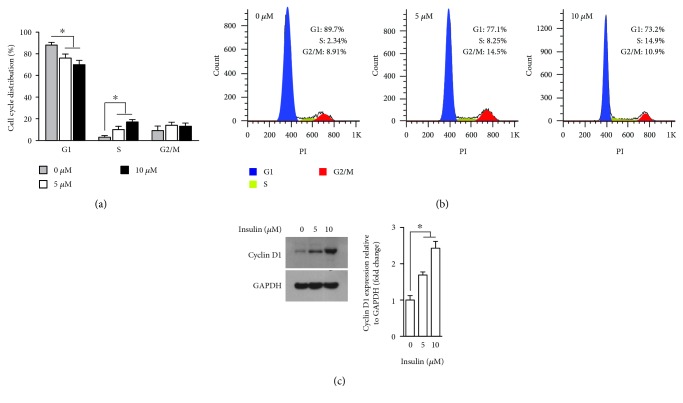
Effects of insulin on the cell cycle distribution and cyclin D1 expression in UCM-MSC. (a, b) UCM-MSC incubated in SFM with 0, 5, and 10 *μ*M insulin for 72 h were subjected to flow cytometry, and the percentages of cells in different cell cycle phases (G1, S, and G2/M) were measured using PI. The bar graph (a) shows the results of three independent experiments and represents the alterations in the cell cycle distribution of UCM-MSC following treatment with the indicated dosages of insulin. Histograms (b) show the data from a representative experiment. (c) UCM-MSC grown in SFM were exposed to 0, 5, and 10 *μ*M insulin for 72 h, and the expression levels of cyclin D1 and GAPDH were assessed by Western blotting. Left, representative blots presenting the protein level of cyclin D1 in UCM-MSC treated with or without the indicated concentrations of insulin (GAPDH served as an internal control for protein loading); right, bar plot showing the cyclin D1/GAPDH ratio, as determined by densitometric analysis of Western blots and expressed as fold change compared with the nontreatment control (*n* = 3). For all panels, data are shown as mean ± SD. ^∗^*p* < 0.05 versus nontreated control cells by one-way ANOVA with Fisher's test.

The proliferation of mammalian cells is tightly coordinated with cell cycle progression [18]; thus, we next determined whether the pro-proliferative effect exerted by relatively high doses of insulin is associated with any changes in the cell cycle distribution. To this end, UCM-MSC exposed to vehicle or insulin (5 and 10 *μ*M) were labeled with a DNA stain and subjected to flow cytometry. DNA content analysis revealed that, while insulin had no significant impact on the G2/M phase distribution, it dose-dependently reduced the percentage of cells in G1 phase with an increased S phase population (Figures 2(a) and 2(b)), a phenomenon generally observed when the G1-S cell cycle transition is accelerated [19]. Cyclin D1 plays a central role in regulating cell proliferation and is critically required for cell cycle progression through the G1 to S stages [20]. Consequently, we examined the expression level of cyclin D1 by Western blotting in UCM-MSC treated with or without insulin. Treatment with 5 and 10 *μ*M of insulin led to a 1.6- and 2.3-fold increase in the cyclin D1 protein level, respectively (Figure 2(c)), confirming the involvement of cyclin D1 in insulin-driven UCM-MSC proliferation and G1-S transition.

### 3.3. Insulin Enhances UCM-MSC Proliferation through Activating the Akt-Cyclin D1 Axis

**Figure 3:**
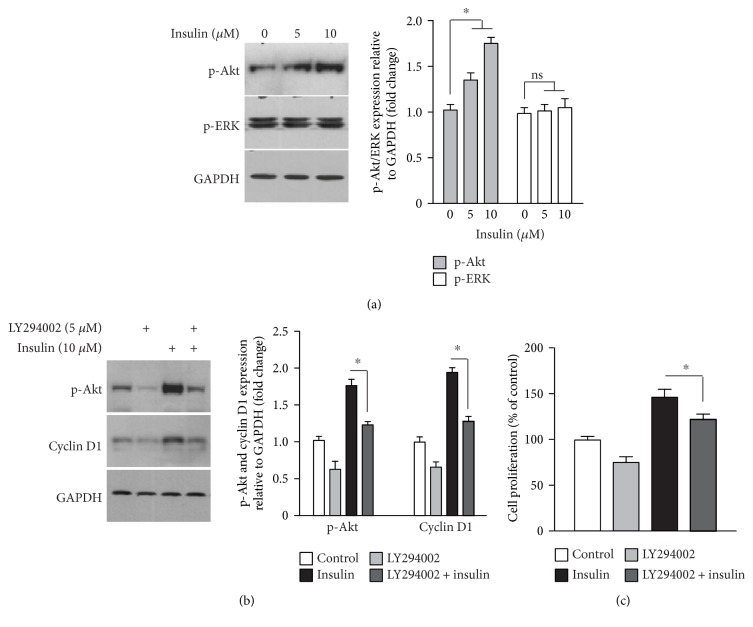
Akt-cyclin D1 pathway activation is responsible for the pro-proliferative effect of insulin in UCM-MSC. (a) Specific activation of Akt in UCM-MSC following insulin treatment. Western blot analysis of phosphorylated Akt and ERK (p-Akt/ERK) in UCM-MSC exposed to 0, 5, and 10 *μ*M insulin for 72 h under serum-deprived conditions. Left, representative blots showing the phosphorylated Akt and ERK protein levels following exposure to the indicated concentrations of insulin (GAPDH served as an internal control for protein loading); right, bar plot showing p-ERK/GAPDH and p-Akt/GAPDH ratios, as evaluated by densitometric analysis of Western blots and normalized to the untreated control. Data are shown as mean ± SD, and error bars indicate SD (*n* = 3). ^∗^*p* < 0.05 versus nontreated control cells by one-way ANOVA with Fisher's test (ns, not significant between indicated groups). (b) Decreases in insulin-induced cyclin D1 and phosphorylated Akt expression in UCM-MSC after Akt inactivation. UCM-MSC cultured in SFM were either left untreated or treated with an Akt inhibitor (LY294002, 5 *μ*M) in the presence and absence of 10 *μ*M insulin for 72 h and then subjected to Western blot analysis with anti-cyclin D1, anti-p-Akt, and anti-GAPDH (loading control) antibodies. Left, representative blots showing the phosphorylated Akt and cyclin D1 protein levels following the indicated treatments; right, bar plots showing p-Akt/GAPDH and cyclin D1/GAPDH ratios as assessed by densitometric analysis of Western blots and normalized to the untreated control. (c) Akt inhibition attenuates the pro-proliferative effect of insulin. UCM-MSC cultured in SFM were subjected to different treatments as described in (b), and cell proliferation was measured by CCK-8 assays. Values are adjusted relative to the proliferation of the nontreatment control, which was set to 100%. For bar graphs in (b) and (c), data are shown as mean ± SD, and error bars indicate SD (*n* = 3). Asterisks denote significant differences between indicated groups by Student's *t*-test (*p* < 0.05).

Cyclin D1-mediated G1-S cell cycle progression in response to growth factors can be modulated by Akt and/or ERK activities [21]. Therefore, we examined the phosphorylated Akt and ERK levels in UCM-MSC treated with or without insulin. The level of phosphorylated ERK remained unaltered by insulin treatment, but the level of Akt phosphorylation was elevated remarkably (Figure 3(a)). This result implies that increased activity of Akt, but not of ERK, is responsible for the cyclin D1-mediated UCM-MSC proliferation induced by insulin. To verify this, UCM-MSC were treated with insulin in the presence or absence of an Akt inhibitor, LY294002, and the effects of insulin on cyclin D1 protein expression as well as cell proliferation were measured. LY294002 markedly attenuated the insulin-induced increases in cyclin D1 and phosphorylated Akt expression levels (Figure 3(b)). Additionally, LY294002 also significantly prevented the increase in cell proliferation stimulated by insulin (Figure 3(c)). These observations together suggest that insulin activates the Akt-cyclin D1 pathway, thereby promoting the proliferation of UCM-MSC.

### 3.4. Insulin Has No Influence on the Immunophenotype and Differentiation Capacity of UCM-MSC

**Figure 4:**
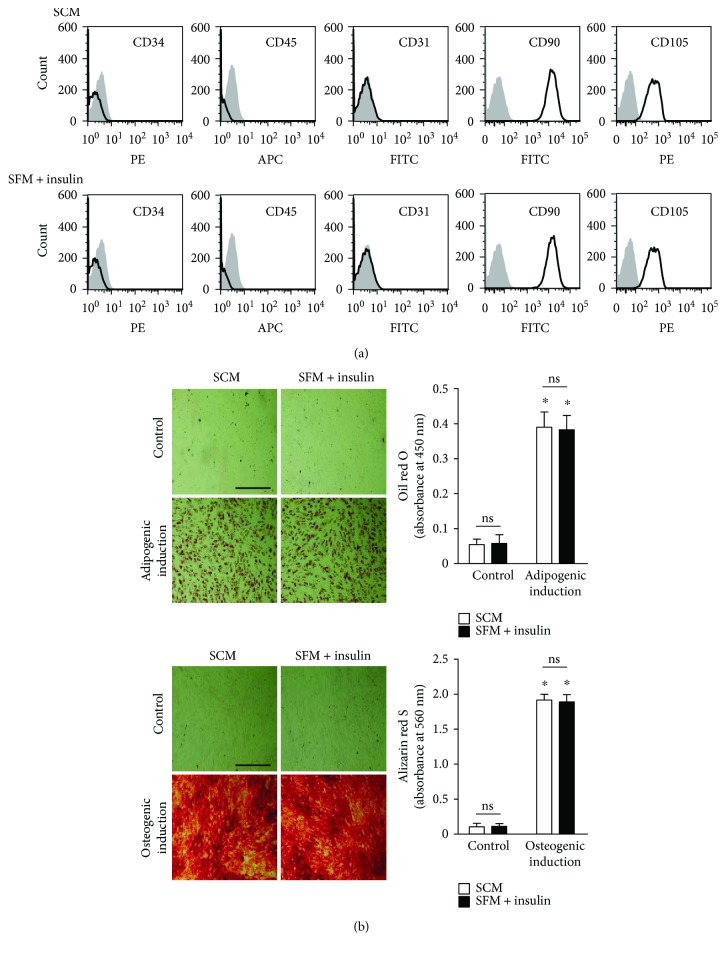
Effects of insulin on the immunophenotype and differentiation capacity of UCM-MSC. (a) UCM-MSC grown in conventional 10% FBS-containing media (SCM) and serum-deficient media supplemented with 10 *μ*M insulin (SFM + insulin) for one passage were analyzed for surface expression of CD34, CD45, CD31, CD90, and CD105 by flow cytometry. Histograms show the data of a representative experiment from three independent studies with similar results (black line: samples; gray filled: corresponding isotype controls). (b) UCM-MSC grown in different conditions over one passage (as described in (a)) were reseeded into 12-well plates. The cells were then left uninduced in SCM (control) or were induced to differentiate into either adipocytes (adipogenic induction) or osteoblasts (osteogenic induction) in appropriate differentiation media for 21 days. After staining the cultures with oil red O or alizarin red S, the cells were photographed under identical brightness and contrast conditions (left), and then the deposited oil red O and alizarin red S were eluted, followed by absorbance measurement at 450 and 560 nm, respectively (right). Scale bar, 500 *μ*m. For both bar graphs, data are shown as mean ± SD, and error bars indicate SD (*n* = 3). ^∗^*p* < 0.05 versus control by one-way ANOVA with Fisher's test (ns: not significant between indicated groups).

The results presented so far indicate that addition of insulin to SFM enhances UCM-MSC proliferation via the Akt-cyclin D1 axis. We next investigated whether UCM-MSC grown under such a condition exhibit any alterations in the expression of surface antigens and differentiation potential. Comparative flow cytometric analysis revealed no noticeable differences in all tested MSC surface markers between cells cultured in SCM and those grown in SFM supplemented with insulin (Figure 4(a)). Furthermore, the results of adipogenic and osteogenic differentiation assays showed that UCM-MSC expanded in SCM and in SFM containing insulin had comparable potentials to differentiate into adipocytes and osteoblasts (Figure 4(b)). These results indicate that adding insulin to SFM does not affect the phenotype and differentiation potential of UCM-MSC.

## 4. Discussion

Consistent with previous studies showing that insulin has pro-proliferative effects on various cell types [12, 14, 22], we demonstrated that insulin at concentrations higher than 2.5 *μ*M dose-dependently increased the number of UCM-MSC in SFM via promoting their proliferation (Figure 1(a)). However, it should be noted that, despite obtaining similar results in UCM-MSC, several investigators reported that insulin at merely nanomolar concentrations significantly increases the proliferation rate of human astrocytes and hepatocytes within 72 h [23, 24], which is inconsistent with our data showing that physiologically relevant concentrations of insulin (0.1–1.0 *μ*M) had no significant effect on UCM-MSC proliferation. One possible explanation for these distinct responses to insulin is the genetic background of different cells. Given that insulin elicits its effect majorly by binding to the insulin receptor (IR) and/or insulin-like growth factor receptor (IGFR) [15], it is possible that relatively low basal expression of IR and IGFR in UCM-MSC might be why they are not responsive to insulin at physiologically relevant dosages. Nevertheless, because there are no data concerning IR and IGFR expression in UCM-MSC, further studies are needed to address whether this is indeed the case.

Based on the observations that UCM-MSC exposed to insulin exhibited elevated cyclin D1 protein expression and an increased S phase population with a parallel decline in the G1 phase population (Figure 2) and that Akt inhibitors abrogated the increases in the cyclin D1 level and cell proliferation induced by insulin (Figure 3), we proposed that insulin enhances UCM-MSC proliferation in SFM by accelerating G1-S transition via activation of the Akt-cyclin D1 axis. Cell cycle progression from G1 to S phase mediates commitment to cell division and is governed by cyclin D1 [20, 25, 26]. As a coregulator of the multiprotein cyclin D1-dependent kinase (CD1K) complex, cyclin D1 activates CD1K and thus drives cells from G1 into S phase via phosphorylation of retinoblastoma protein (Rb) and subsequent release of Rb-bound E2F transcription factors [27, 28]. Although acceleration of G1-S transition represents one well-defined mechanism by which cyclin D1 promotes cell proliferation, results from prior studies imply that alternative pathways also exist [29]. For example, Albrecht et al. [30] reported that enhanced proliferation of hepatocytes caused by cyclin D1 over-expression is attributed to augmented cell growth. Similarly, increased cell growth was accompanied by insulin-induced cyclin D1 expression and UCM-MSC proliferation in our study (Figure 1(c)). Therefore, we cannot exclude the possibility that the enhanced growth of UCM-MSC also might be mediated by cyclin D1 and this somehow contributes to the pro-proliferative effect of insulin. In support of this view, insulin is a potent activator of mammalian target of rapamycin kinase, which not only functions as an upstream modulator of cyclin D1 but also is capable of simultaneously regulating both cell growth and proliferation [30, 31].

Although UCM-MSC are a promising candidate for treatment of a variety of disorders, how to obtain sufficient numbers of UCM-MSC under serum-deficient conditions remains an obstacle [4, 5]. Our work revealed that the immunological characteristics and differentiation capacities of UCM-MSC propagated in SFM containing insulin and conventional SCM were comparable (Figure 4). This finding, together with the result that insulin promotes UCM-MSC proliferation in SFM, thus has important implications for developing future strategies to use insulin in clinical-grade production of UCM-MSC. Several chemically defined serum-free media such as StemPro® MSC SFM from Invitrogen and MesenCult™-XF from Stem Cell Technologies are now commercially available for MSC expansion. Yet, they seem to generate MSC with different characteristics (e.g., growth pattern, phenotype, and differentiation potentials) compared with those cultured in conventional FBS-based media, indicating that the performance of these commercial media on MSC growth is questionable and the medium formulations lack some known and unknown factors included in serum [32]. Insulin is an important component of FBS and plays a key role in cellular metabolism [7, 33]. Therefore, it is reasonable to assume that in addition to other growth-promoting constituents provided by FBS, an ideal serum-deficient medium should also consist of insulin. However, as most commercial media formulations including the abovementioned ones are not disclosed, whether these media are supplemented with insulin is unclear. It came to our notice that Jung et al. [32, 34] have developed a serum-free medium (PPRF-msc6) for MSC expansion with a disclosed formulation. They showed that in comparison to MSC cultured in currently available commercial media and conventional SCM, the cells grown in PPRF-msc6 had a much shorter doubling time and generated more colony-forming units. It deserves to be mentioned that insulin is included in their medium formulation and utilized at a concentration of 3.4 *μ*M, thereby supporting the results reported herein.

Notably, because insulin is routinely employed as a component of adipogenic differentiation medium in vitro [35], it at first sight appears paradoxical that the adipogenic differentiation potential of UCM-MSC generated in SFM containing insulin was unaltered compared with that of UCM-MSC cultured in SCM. However, we and other groups have previously demonstrated that insulin acts solely to accelerate the process of lipid filling at late stages of adipocyte maturation and does not influence the onset of adipogenesis (e.g., adipogenic commitment of BM-MSC) [36, 37]. In light of this, it is thus not surprising that the adipogenic potential of UCM-MSC generated in SFM containing insulin remained unchanged.

## 5. Conclusions

In conclusion, our results revealed that high doses of insulin (higher than physiologically relevant concentrations) enhanced UCM-MSC proliferation under serum-deficient conditions by activation of the Akt-cyclin D1 axis, without affecting their immunophenotype and differentiation capacities. These findings highlight the emerging functional role of insulin signaling in regulating UCM-MSC division and have important implications for in vitro expansion of UCM-MSC prior to their clinical applications.

## Supplementary Material

Supplementary Figure 1. The long-term effects of insulin on MSC viability under SCM and SFM conditions.

## Abbreviations

APC: Allophycocyanin
BM: Bone marrow
CCK-8: Cell-counting kit-8
CD1K: Cyclin D1-dependent kinase
ERK: Extracellular signal-regulated kinase
FBS: Fetal bovine serum
FITC: Fluorescein isothiocyanate
GAPDH: Glyceraldehyde-3-phosphate dehydrogenase
IGFR: Insulin-like growth factor receptor
IR: Insulin receptor
MSC: Mesenchymal stem cells
PBS: Phosphate-buffered saline
PE: Phycoerythrin
PI: Propidium iodide
Rb: Retinoblastoma protein
SCM: Serum-containing media
SFM: Serum-free medium
UCM: Umbilical cord matrix.

## Additional Points

*Availability of Data and Material*. All data generated or analyzed during this study are included in this manuscript.
