# Correction to “Insulin Promotes the Proliferation of Human Umbilical Cord Matrix‐Derived Mesenchymal Stem Cells by Activating the Akt‐Cyclin D1 Axis”

**DOI:** 10.1155/sci/9879105

**Published:** 2026-07-22

P. Li, J. Wei, X. Gao, et al., “Insulin Promotes the Proliferation of Human Umbilical Cord Matrix‐Derived Mesenchymal Stem Cells by Activating the Akt‐Cyclin D1 Axis” *Stem Cells International*, vol. 2017 (2017). https://doi.org/10.1155/2017/7371615.

In the article titled “Insulin Promotes the Proliferation of Human Umbilical Cord Matrix‐Derived Mesenchymal Stem Cells by Activating the Akt‐Cyclin D1 Axis,” there was an error in Figure 4b. More specifically, the panel representing the cells from the adipogenic induction group grown in SCM shared overlapping features with the adjacent panel, which depicted the cells from the same group grown under different conditions. This error was introduced by the authors during manuscript preparation assembly, and the corrected Figure 4 is shown below:

Figure 4Effects of insulin on the immunophenotype and differentiation capacity of UCM‐MSC. (a) UCM‐MSC grown in conventional 10%FBS‐containing media (SCM) and serum‐deficient media supplemented with 10 μM insulin (SFM + insulin) for one passage were analyzed for surface expression of CD34, CD45, CD31, CD90, and CD105 by flow cytometry. Histograms show the data of a representative experiment from three independent studies with similar results (black line: samples; gray filled: corresponding isotype controls). (b) UCM‐MSC grown in different conditions over one passage (as described in (a)) were reseeded into 12‐well plates. The cells were then left uninduced in SCM (control) or were induced to differentiate into either adipocytes (adipogenic induction) or osteoblasts (osteogenic induction) inappropriate differentiation media for 21 days. After staining the cultures with oil red O or alizarin red S, the cells were photographed under identical brightness and contrast conditions (b1), and then the deposited oil red O and alizarin red S were eluted, followed by absorbance measurement at 450 and 560 nm, respectively (b2). Scale bar, 500 μm. For both bar graphs, data are shown as mean ± SD, and error bars indicate SD (*n* = 3).  ^∗^ *p* < 005 versus control by one‐way ANOVA with Fisher’s test (ns: not significant between indicated groups).

**Figure:**
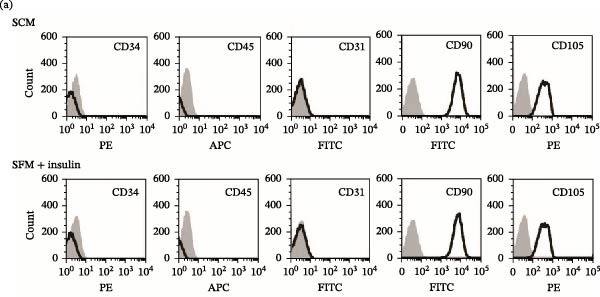

**Figure:**
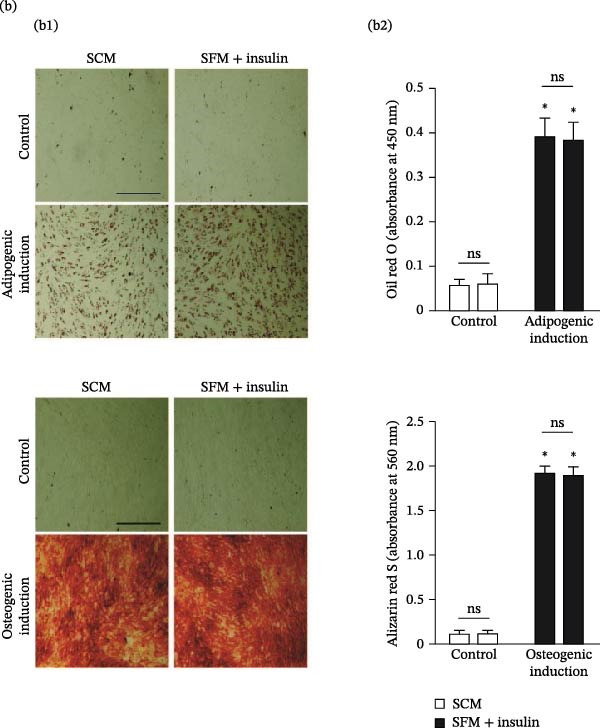

We apologize for this error.

