# Supplementary material for: Insulin Promotes the Proliferation of Human Umbilical Cord Matrix-Derived Mesenchymal Stem Cells by Activating the Akt-Cyclin D1 Axis
Source: Stem Cells Int. 2017 Apr 18;2017:7371615. doi: 10.1155/2017/7371615 (PMC5412176; doi:10.1155/2017/7371615)
Supplement: Supplementary file 1 — Supplementary Figure 1. The long-term effects of insulin on MSC viability under SCM and SFM conditions. [file 7371615.f1.docx]

**Supplementary information**

**Insulin promotes the proliferation of human umbilical cord matrix-derived mesenchymal stem cells by activating the Akt-cyclin D1 axis**

Peng Li^a,^**^#^**, Jinsong Wei^b,^**^#^** , Xiang Gao^a^, Bo Wei^b^, Hao Lin^b^, Rui Huang^a^, Yanru Niu^c^, Kyu Lim^d^, Kaipeng Jing^a,c,^***** and Jiaqi Chu^a,b,^*****

^a^Stem Cell Research and Cellular Therapy Center, and ^b^Department of Spinal Surgery, and ^c^Laboratory Institute of Minimally Invasive Orthopedic Surgery, Affiliated Hospital of Guangdong Medical University, Zhanjiang, China; ^d^Department of Biochemistry, School of Medicine, Chungnam National University, Daejeon, Korea

**Supplementary Figure 1**


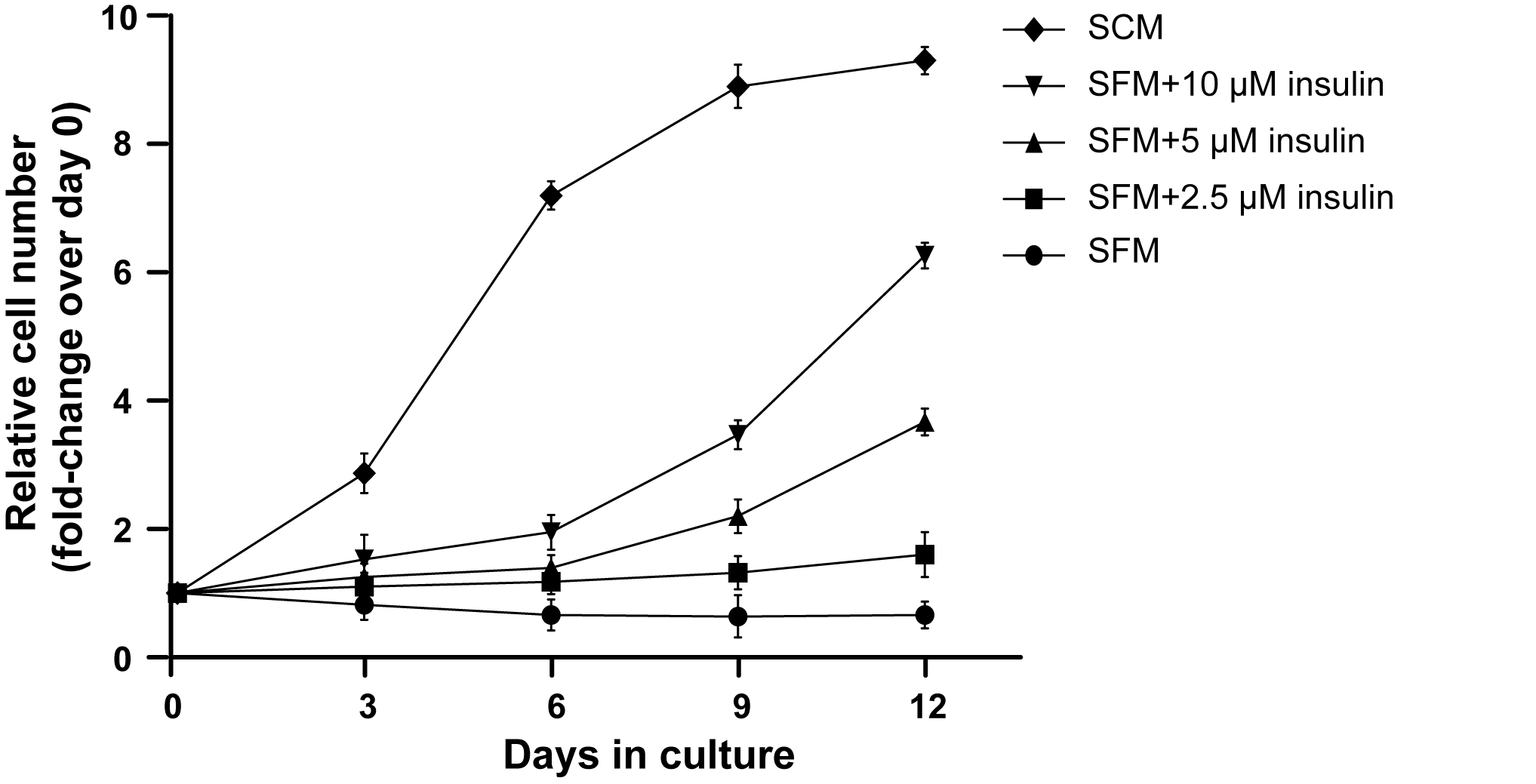


**Supplementary Figure 1**. In vitro growth curve of UCM-MSC in serum-free medium (SFM), serum-containing medium (SCM) and SFM supplemented with various concentrations of insulin. UCM-MSC suspended in SCM were seeded into 96-well plates at 2000 cells per well. After an overnight incubation to allow attachment (day 0), the cells were washed once with warm PBS and shifted to each indicated condition, and the proliferation of cells was assessed at five different time points (day 0, 3, 6, 9 and 12) by the CCK-8 assay. Relative cell number expressed as fold-change relative to the cell counts initially plated on day 0 is shown. All data are represented as mean ± SD, and error bars indicate SD (n=3).
